# Metformin Therapy Changes Gut Microbiota Alpha-Diversity in COVID-19 Patients with Type 2 Diabetes: The Role of SARS-CoV-2 Variants and Antibiotic Treatment

**DOI:** 10.3390/ph16060904

**Published:** 2023-06-20

**Authors:** Pavlo Petakh, Iryna Kamyshna, Valentyn Oksenych, Denis Kainov, Aleksandr Kamyshnyi

**Affiliations:** 1 Department of Biochemistry and Pharmacology, Uzhhorod National University, 88000 Uzhhorod, Ukraine; 2 Department of Microbiology, Virology, and Immunology, I. Horbachevsky Ternopil National Medical University, 46001 Ternopil, Ukraine; 3 Department of Medical Rehabilitation, I. Horbachevsky Ternopil National Medical University, 46001 Ternopil, Ukraine; 4 Broegelmann Research Laboratory, Department of Clinical Science, University of Bergen, 5020 Bergen, Norway; 5 Department for Clinical and Molecular Medicine (IKOM), Norwegian University of Science and Technology, 7491 Trondheim, Norway; 6 Institute of Technology, University of Tartu, 50090 Tartu, Estonia

**Keywords:** COVID-19, gut microbiota, diversity, diabetes, metformin

## Abstract

The gut microbiota play a crucial role in maintaining host health and have a significant impact on human health and disease. In this study, we investigated the alpha diversity of gut microbiota in COVID-19 patients and analyzed the impact of COVID-19 variants, antibiotic treatment, type 2 diabetes (T2D), and metformin therapy on gut microbiota composition and diversity. We used a culture-based method to analyze the gut microbiota and calculated alpha-diversity using the Shannon H′ and Simpson 1/D indices. We collected clinical data, such as the length of hospital stay (LoS), C-reactive protein (CRP) levels, and neutrophil-to-lymphocyte ratio. We found that patients with T2D had significantly lower alpha-diversity than those without T2D. Antibiotic use was associated with a reduction in alpha-diversity, while metformin therapy was associated with an increase. We did not find significant differences in alpha-diversity between the Delta and Omicron groups. The length of hospital stay, CRP levels, and NLR showed weak to moderate correlations with alpha diversity. Our findings suggest that maintaining a diverse gut microbiota may benefit COVID-19 patients with T2D. Interventions to preserve or restore gut microbiota diversity, such as avoiding unnecessary antibiotic use, promoting metformin therapy, and incorporating probiotics, may improve patient outcomes.

## 1. Introduction

The gut microbiota is a complex and diverse community of microorganisms that reside in the gastrointestinal tract [1]. It plays a crucial role in the maintenance of the host’s health and wellbeing by performing important metabolic, immunological, and nutritional functions [2]. Recent studies have shown that alterations in the composition and diversity of gut microbiota can have a significant impact on human health and disease [3].

The gut microbiota have been shown to play a crucial role in regulating glucose metabolism and overall metabolic health in individuals with type 2 diabetes (T2D). Several studies have found that individuals with T2D have a less diverse gut microbiota, with a lower abundance of beneficial bacteria, such as *Bifidobacterium* spp. and *Akkermansia muciniphila*, and a higher abundance of harmful bacteria, such as *Firmicutes* and *Proteobacteria* [4,5,6,7,8].

This dysbiosis in the gut microbiota has been linked to impaired glucose metabolism and insulin resistance in individuals with T2D [9]. The gut microbiota is involved in the production of short-chain fatty acids (SCFAs), which are important for glucose and lipid metabolism [10]. SCFAs can promote the release of insulin and improve glucose uptake by peripheral tissues [11]. Additionally, the gut microbiota can influence host metabolism through the production of various metabolites, such as bile acids, which are involved in regulating glucose and lipid metabolism [12].

Metformin therapy, a commonly used drug for the treatment of T2D, has been found to impact the gut microbiota by increasing the abundance of beneficial bacteria, such as *Akkermansia muciniphila* and *Faecalibacterium prausnitzii*, while reducing the abundance of harmful bacteria such as *Escherichia coli* [13,14]. This suggests that metformin may improve glucose metabolism in individuals with T2D by modulating the gut microbiota [15].

The COVID-19 pandemic has brought the importance of gut microbiota into the spotlight, as research has suggested that COVID-19 patients with gastrointestinal symptoms may have a different gut microbiota composition compared to those without such symptoms [16,17,18,19,20,21]. Additionally, individuals with pre-existing conditions such as T2D have been found to have less diverse gut microbiota, which may contribute to the progression and severity of COVID-19 [22].

Antibiotic treatment is commonly used to manage bacterial infections, but it can also affect the gut microbiota by altering their composition and reducing their diversity [23]. Understanding the impact of COVID-19, antibiotic treatment, metformin therapy, and other factors on gut microbiota composition and diversity could provide valuable insights into the pathogenesis and management of COVID-19 in patients with T2D, and may lead to the development of targeted interventions aimed at modulating the gut microbiota to improve patient outcomes. In this study, we aimed to investigate the alpha diversity of gut microbiota in patients with COVID-19 and T2D, and to analyze the impact of these factors on the gut microbiota composition and diversity. We used a culture-based method to analyze the gut microbiota, which enabled us to identify and quantify bacterial species present in the gut.

## 2. Results

**Figure 1:**
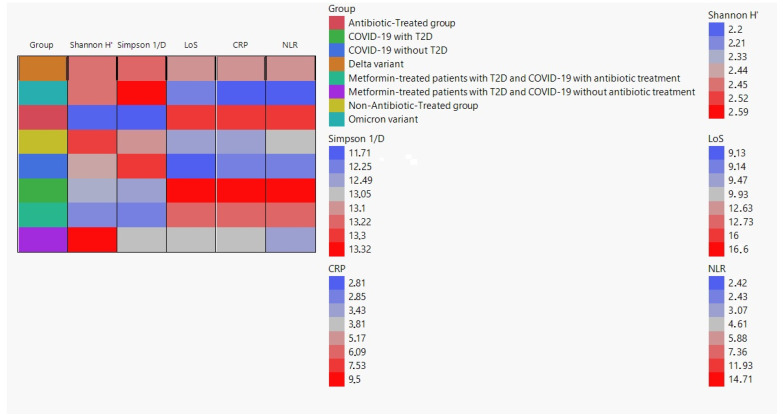
Cell plot of alpha diversity of gut microbiota (Simpson 1/D and Shannon H′) in COVID-19 patients and clinical parameters, including length of hospital stay (LoS), C-Reactive Protein (CRP) Levels, and Neutrophil-to-Lymphocyte Ratio (NLR).

We created a cell plot to visualize the average values of Shannon H′, Simpson 1/D, LoS (length of hospital stay), CRP (C-Reactive Protein), and NLR (Neutrophil-to-Lymphocyte Ratio) among different groups. The data used for the cell plot was obtained from the following groups: Delta variant, Omicron variant, antibiotic-treated group, non-antibiotic-treated group, COVID-19 without T2D, COVID-19 with T2D, metformin-treated patients with T2D and COVID-19 with antibiotic treatment, and metformin-treated patients with T2D and COVID-19 without antibiotic treatment (Figure 1).

### 2.1. Patient Population

The patient population of this study included 120 COVID-19-confirmed patients who were admitted to the Transcarpathian Regional Infectious Hospital from 2020 to 2022 and were assigned to eight different study groups. The demographic characteristics of the patients in each study group are summarized in Table 1. Overall, the mean age of the patients was 54.1 ± 9.5 years, with a range of 20 to 80 years. Both female (51.7%) and male (48.3%) patients were included in the study.

The age of patients varied across the different study groups. The mean age of patients in the Delta variant (Group A), Omicron variant (Group B), and antibiotic-treated groups (Group C), was similar (55.1 ± 11.4, 56.7 ± 4.9, and 55.3 ± 11.8 years, respectively). In contrast, patients in the Non-Antibiotic-Treated group (Group D) were significantly younger, with a mean age of 43.6 ± 9.7 years. Patients with T2D were also older, with a mean age of 61.5 ± 8.0 years in the COVID-19 with T2D group.

Gender distribution was similar across all study groups, with no significant differences observed between males and females. However, the “metformin-treated patients with T2D and COVID-19 with antibiotic treatment” group (Group G) had a different gender distribution, with 53.4% of patients being male and 46.6% female.

Overall, the study population was representative of the intended patient population, with patients from different age groups and both genders included in the study.

### 2.2. Alpha-Diversity Analysis

**Figure 2:**
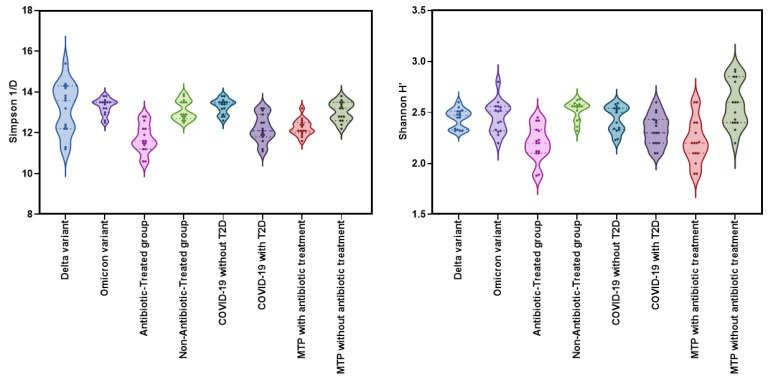
Violin plot depicting the distribution of alpha diversity indices (Shannon H′ and Simpson 1/D) among eight patient groups.

The Kruskal–Wallis test was used to compare the alpha diversity indices (Shannon H′ and Simpson 1/D) among eight groups: (1) Delta variant (Group A), (2) Omicron variant (Group B), (3) antibiotic-treated group (Group C), (4) Non-antibiotic-treated group (Group D), (5) COVID-19 without T2D (Group E), (6) COVID-19 with T2D (Group F), (7) metformin-treated patients with T2D and COVID-19 with antibiotic treatment (Group G), and (8) metformin-treated patients with T2D and COVID-19 without antibiotic treatment (Group H). The results showed significant differences among the groups for both Shannon H′ (χ^2^ = 45.3, df = 9, *p* < 0.001, ε^2^ = 0.381) and Simpson 1/D (χ^2^ = 57.3, df = 9, *p* < 0.001, ε^2^ = 0.482) (Figure 2). The mean and standard deviation values for each group are shown in Table 2.

The Shannon H index did not show any significant differences in alpha-diversity between the Delta and Omicron variant groups (W = 0.588; *p* = 1.000). The antibiotic-treated group had significantly lower diversity (W = 5.995; *p* < 0.001) compared to the non-antibiotic-treated group. However, there was no significant difference in the Shannon H index (*p* = 0.544) between COVID-19 patients with and without T2D, suggesting that the presence of T2D may not significantly affect alpha-diversity in patients with COVID-19 and T2D.

**Figure 3:**
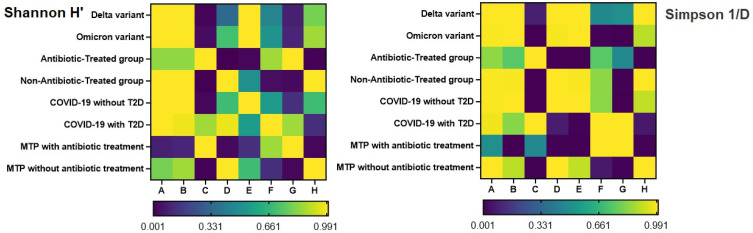
Heatmap of Dwass–Steel–Critchlow–Fligner (DSCF) post hoc test p-values for comparison of alpha diversity indices among COVID-19 and T2D patient groups and treatments. The *p*-values are color-coded, with higher values displayed in lighter colors and lower values in darker colors. (Group A—Delta variant; Group B—Omicron variant; Group C—antibiotic-treated group; Group D—non-antibiotic-treated group; Group E—COVID-19 without T2D; Group F—COVID-19 with T2D; Group G—metformin-treated patients with T2D and COVID-19 with antibiotic treatment; Group H—metformin-treated patients with T2D and COVID-19 without antibiotic treatment).

Significant differences were observed in Shannon H′ index (W = 5.017, *p* = 0.014) between “Metformin-treated patients with T2D and COVID-19 with antibiotic treatment (Group G)” and “Metformin-treated patients with T2D and COVID-19 without antibiotic treatment (Group H)” groups. The DSCF test revealed a significant difference in Simpson 1/d (W = 5.913; *p* < 0.001) between the “Antibiotic-Treated” group and the “Non-Antibiotic-Treated” group. The DSCF test also indicated a significant difference in Simpson 1/d (*p* = 0.006) between “Metformin-treated patients with T2D and COVID-19 with antibiotic treatment” as well as “Metformin-treated patients with T2D and COVID-19 without antibiotic treatment” groups. Furthermore, significant differences in Simpson 1/d were observed between “COVID-19 patients without T2D” (Group E) and those with T2D (Group F) (W = −5.352; *p* = 0.006) (Figure 3).

### 2.3. Correlation between Alpha Diversity Indices and Clinical Parameters

In our study, we conducted a correlation analysis between alpha diversity indices (Shannon H′ and Simpson 1/D), LoS, CRP, and NLR in our patient population. Spearman rank correlation was used to analyze the data, and the results are presented in the correlation matrix.

**Figure 4:**
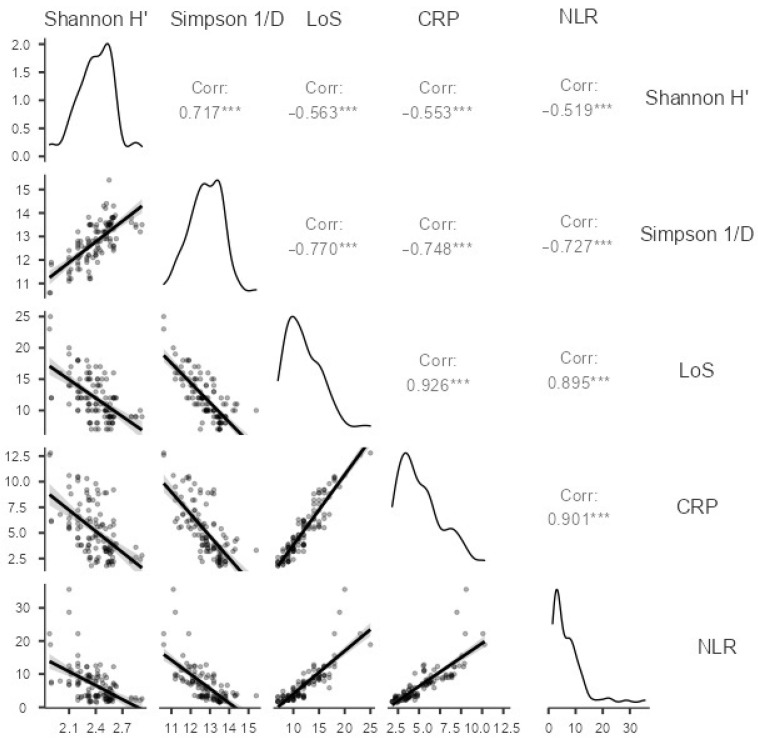
Correlation matrix analysis of alpha diversity indices, length of stay (LoS), C-reactive protein (CRP), and neutrophil-to-lymphocyte ratio (NLR) in COVID-19 patients. Statistical significance of correlations *** *p* < 0.005. Grey zones indicate 95% confidence intervals.

The Shannon H′ index showed a significant positive correlation with the Simpson 1/D index (Spearman’s rho = 0.717, *p* < 0.001). Additionally, there was a significant negative correlation between the Shannon H′ index and all three clinical variables: LoS (Spearman’s rho = −0.563, *p* < 0.001), CRP (r = −0.553, *p* < 0.001), and NLR (r = −0.519, *p* < 0.001). The Simpson 1/D index showed a similar negative correlation with LoS, CRP, and NLR (Spearman’s rho ranged from −0.727 to −0.748, all *p* < 0.001) (Figure 4).

These findings suggest that decreased alpha diversity is associated with increased LoS, higher levels of CRP, and a higher NLR in our patient population. These correlations may provide insight into the underlying mechanisms and clinical implications of altered gut microbiota in COVID-19 patients.

### 2.4. Binary Logistic Regression

A predictive model was developed to estimate the probability of COVID-19 with T2D, conditioning on Shannon H′ and Simpson 1/D, using binary logistic regression.

$$P=\frac{1}{1+e^{-z}}\times 100\%,z=46.959+12.787\times ShannonH\prime -5.989\times Simpson\;1/D$$

**Figure 5:**
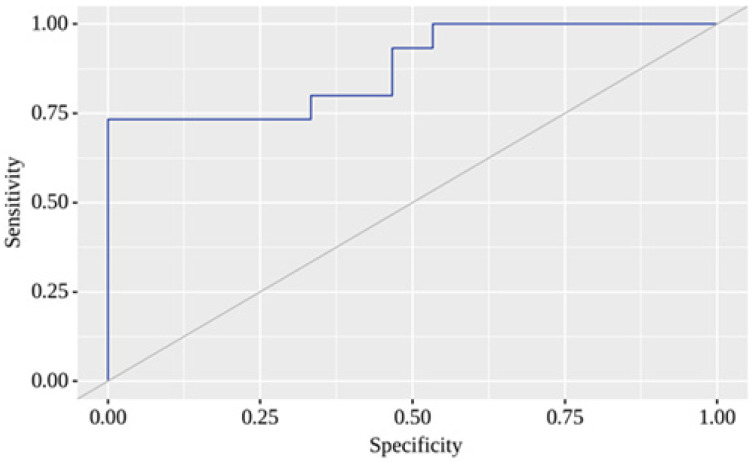
ROC analysis of a predictive model for estimating COVID-19 probability with T2D based on Shannon H′ and Simpson 1/D. The sensitivity and specificity for the curve were 73.3% and 100%, respectively. The area under the ROC curve comprised 0.880 ± 0.065 with 95% CI: 0.752–1.000. The resulting model was statistically significant (*p* < 0.001). The diagonal line (grey line) denotes the ROC curve of a random classifier.

Here, P represents the probability of a positive result. The resulting regression model was statistically significant (*p* < 0.001). Based on the Nagelkerke R² value, the model explains 59.1% of the observed COVID-19 with T2D variance. When evaluating the dependence of the probability of a positive result on the value of the logistic function P using ROC analysis, the resulting curve had an area under the ROC curve of 0.880 ± 0.065 with 95% CI: 0.752–1.000 (Figure 5).

**Figure 6:**
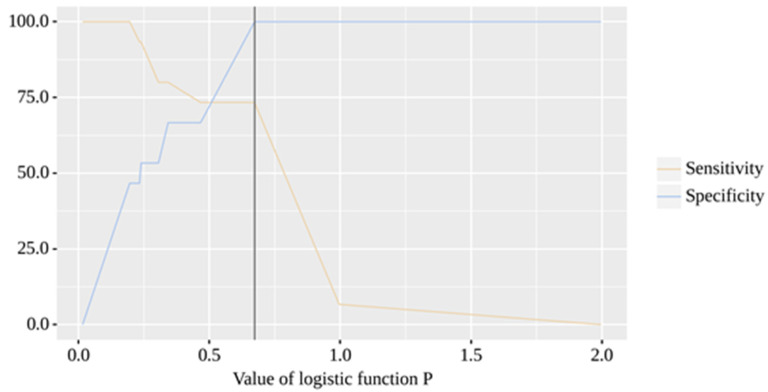
Sensitivity and specificity analysis of COVID-19 with T2D using logistic function.

The resulting model was statistically significant (*p* < 0.001). The cut-off value of the logistic function P, which corresponds to the highest Youden’s J statistic, is 0.673. If the value of the logistic function P was greater than or equal to this value, a positive result was predicted. The sensitivity and specificity of the method were 73.3% and 100.0%, respectively (Figure 6).

## 3. Discussion

In this study, we investigated alterations in gut microbiota diversity in patients with COVID-19 and T2D, as well as the potential association between gut microbiota diversity and clinical parameters. Our findings indicate that the use of antibiotics and the presence of T2D in COVID-19 patients may have an impact on gut microbiota diversity, as demonstrated by the significant differences in alpha diversity indices observed among the eight groups. Specifically, the “Antibiotic-Treated” group (Group C) had significantly lower diversity compared to the “Non-Antibiotic-Treated” group (Group D), and there was no significant difference in the Shannon H index between “COVID-19 without T2D” (Group E) and “COVID-19 with T2D” (Group F), suggesting that T2D may not significantly affect alpha diversity in “COVID-19 patients with T2D” (Group F). The findings from our study reveal that there are significant differences in alpha-diversity (Shannon H) between “metformin-treated patients with T2D and COVID-19 with antibiotic treatment and metformin-treated patients with T2D” (Group G) and “COVID-19 without antibiotic treatment” (Group H). Specifically, the alpha-diversity was found to be higher in the group of patients who did not receive antibiotic treatment (Group H) compared to those who did (Group G).

This observation is of significant interest as it suggests that the use of antibiotics may have a detrimental effect on the gut microbiome in individuals with T2D and COVID-19 who are being treated with metformin. Previous studies have demonstrated the importance of a healthy gut microbiome in regulating glucose metabolism and maintaining overall metabolic health in individuals with T2D. Therefore, the observed differences in alpha-diversity between the two groups of patients may have important clinical implications. Our findings are consistent with those reported by other researchers who have investigated the impact of antibiotics on gut microbiota diversity in various patient populations, including COVID-19 patients [24,25,26,27,28].

Our study also revealed a significant positive correlation between the Shannon H′ and Simpson 1/D indices and a significant negative correlation between these indices and clinical parameters such as LoS, CRP, and NLR in our patient population. These findings suggest that decreased alpha diversity is associated with increased LoS, higher levels of CRP, and a higher NLR in our patient population. This is in line with previous studies that have reported similar correlations between gut microbiota and clinical parameters such as disease severity, inflammation, and immune response in various patient populations, including COVID-19 patients [22,29]. Furthermore, our predictive model, which estimates the probability of COVID-19 with T2D based on Shannon H′ and Simpson 1/D, demonstrated good predictive accuracy, with an area under the ROC curve of 0.880.

Previous studies have shown the importance of a healthy gut microbiome in regulating glucose metabolism and maintaining overall metabolic health in individuals with T2D. The gut microbiota plays a crucial role in glucose metabolism by producing SCFAs, such as butyrate, which can improve insulin sensitivity and reduce inflammation in the body [30]. Certain bacterial strains can also produce incretin hormones that stimulate insulin secretion, while others can produce bioactive compounds that affect glucose absorption in the gut [31]. Therefore, alterations in gut microbiota composition and diversity can lead to dysregulation of glucose metabolism and contribute to the development and progression of T2D. 

Dysbiosis of the gut microbiota, characterized by a decrease in SCFA-producing bacteria and an increase in pro-inflammatory bacteria, has been linked to impaired glucose metabolism and the development of T2D [32]. Antibiotic treatment has been shown to reduce microbial diversity and alter the composition of the gut microbiota [23,24,33,34,35]. In individuals with T2D, antibiotic treatment has been associated with a decrease in SCFA-producing bacteria, including *Faecalibacterium prausnitzii* and *Bifidobacterium* spp., and an increase in potentially harmful bacteria, including *Enterobacteriaceae* spp. and *Clostridium difficile* [36,37,38]. These changes in gut microbiota composition and function could potentially contribute to impaired glucose metabolism and the development of T2D. Furthermore, it is well-established that there is a link between gut microbiota and dietary consumption, and dietary changes can lead to alterations in gut microbiota composition [39]. Therefore, it is possible that changes in dietary habits of COVID-19 patients with T2D, either due to illness or hospitalization, may have contributed to the observed changes in gut microbiota diversity. 

Another important factor to consider is the use of metformin, a common medication used to manage T2D. Metformin has been shown not only to modulate gut microbiota but also to reduce oxidative stress, a key contributor to the development and progression of T2D, by activating the enzyme AMP-activated protein kinase (AMPK), which leads to the increased production of antioxidants and decreased production of reactive oxygen species (ROS) [40,41,42]. This mechanism has been well-studied in the context of T2D, but its potential effects on the gut microbiota are less clear. It has also been suggested that metformin may have a beneficial effect in patients with COVID-19, as it has been shown to reduce inflammation and improve lung function in preclinical models of respiratory infections [43].

In conclusion, our study provides evidence for the potential impact of antibiotics and T2D on gut microbiota diversity in COVID-19 patients and the potential association between gut microbiota diversity and clinical parameters [44,45,46,47,48,49]. The findings of this study have several potential clinical implications. Firstly, the observed correlations between gut microbiota diversity and clinical parameters such as LoS, CRP, and NLR in COVID-19 patients with T2D suggest that monitoring gut microbiota diversity may serve as a non-invasive biomarker for disease severity and the response to treatment. Therefore, future studies should investigate the potential of gut microbiota diversity as a prognostic tool for COVID-19 patients with T2D. Secondly, the predictive model developed in this study may have implications for the development of personalized therapeutic strategies for COVID-19 patients with T2D. The model can potentially be used to identify patients who are at a high risk of developing severe disease and guide treatment decisions accordingly. Future research should aim to validate this model in larger patient populations and investigate its potential for use in clinical practice. Lastly, the observed association between metformin treatment and gut microbiota diversity in COVID-19 patients with T2D is an important finding that warrants further investigation. Metformin is a commonly prescribed medication for the management of T2D and has been shown to have anti-inflammatory effects. In this study, the group of metformin-treated patients with T2D and COVID-19 without antibiotic treatment had a significantly higher gut microbiota diversity compared to the group of metformin-treated patients with T2D and COVID-19 with antibiotic treatment.

Overall, the findings of this study highlight the importance of considering the gut microbiota in the management of COVID-19 patients with T2D and provide a basis for future research in this area. These findings may inform the development of personalized therapeutic strategies for COVID-19 patients with T2D that target the gut microbiota and inflammation biomarkers. Further studies are needed to elucidate the mechanisms underlying the observed associations and to investigate the potential of gut microbiota modulation as a therapeutic strategy for COVID-19 patients with T2D.

## 4. Material and Methods

### 4.1. Study Design and Sample Collection

**Figure 7:**
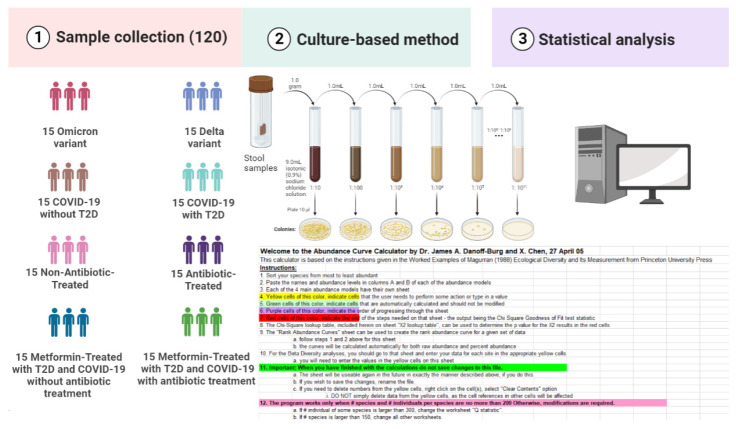
Study flow diagram: (1) sample collection. The study was conducted on 120 fecal samples. (2) Microbiota analysis (culture-based method). (3) Calculation of alpha diversity indices and statistical analysis (using the abundance curve calculator).

The study included 120 fecal samples collected from COVID-19-confirmed patients admitted to the Transcarpathian Regional Infectious Hospital from 2020 to 2022. The patients were assigned to different groups based on specific criteria. The groups included the Delta variant, Omicron variant, an antibiotic-treated group, a non-antibiotic-treated group, COVID-19 without T2D, COVID-19 with T2D, metformin-treated patients (MTP) with T2D and COVID-19 with antibiotic treatment, and metformin-treated patients with T2D and COVID-19 without antibiotic treatment (Figure 7).

Patients were assigned to different groups based on specific criteria. The Delta (Group A) and Omicron (Group B) groups were assigned based on the predominant strains of SARS-CoV-2 during the study period. The antibiotic-treated group (Group C) received linezolid, meropenem, fluoroquinolones (moxifloxacin and ciprofloxacin), or cephalosporins of the III or IV generations during their hospital stay. The non-antibiotic-treated group (Group D), consisting of patients with COVID-19, did not receive any antibiotics during their hospitalization.

The COVID-19 without T2D group (Group E) included patients who tested positive for COVID-19 but did not have T2D. The COVID-19 with T2D group (Group F) included patients who tested positive for both COVID-19 and T2D. These patients were treated with glucose-lowering medications as needed, including metformin, insulin, or other drugs.

The metformin-treated patients (MTP) with T2D and COVID-19 with antibiotic treatment group (Group G) included patients with T2D and COVID-19 who were treated with metformin and received antibiotic treatment during their hospital stay. The metformin-treated patients with T2D and COVID-19 without antibiotic treatment group (Group H) included patients with T2D and COVID-19 who were treated with metformin but did not receive antibiotic treatment during their hospital stay. Metformin-treated patients took a daily dose of 1000–1500 mg of metformin for at least 3 months prior to admission.

The primary COVID-19 diagnostic method is the real-time reverse transcription–polymerase chain reaction (RT–PCR) test, which detects the presence of SARS-CoV-2 viral RNA in respiratory specimens. For T2D, diagnosis is based on medical history. Other diagnostic parameters, such as physical examination and blood glucose tests, may have also been used to confirm the diagnosis.

To ensure adequate statistical power, a power analysis was conducted to justify the sample size used in this study. Based on previous studies and assuming an effect size of 0.5 with a two-sided alpha of 0.05, a sample size of at least 100 was required to achieve 80% power. Therefore, a sample size of 120 was chosen to ensure adequate power and account for potential dropouts or missing data.

### 4.2. Microbiota Analysis and Calculation of Alpha-Diversity

For gut microbiota analysis, the weight of the fecal sample (1.0 g) was recorded, and 9 mL of isotonic (0.9%) sodium chloride solution was added to a test tube. The mixture was thoroughly rubbed until a homogeneous mass was formed, creating a 10^−1^ dilution. Subsequently, a series of dilutions from 10^−2^ to 10^−11^ were prepared in the same way (Figure 7). Using sterile micropipettes, 10 μL was taken from each dilution and applied to nutrient media for the isolation of specific microorganisms. Commercial nutrient media was used for the isolation of enterobacteria, *Staphylococcus* spp., *Enterococcus* spp., yeast (*Candida* spp.), *Clostridium* spp., *Lactobacillus* spp., *Bifidobacterium* spp., and *Bacteroides* spp. The identification of microorganisms was carried out based on the Clinical Microbiology Procedures Handbook, Volume 1–3, 4th Edition [50]. Decimal logarithms of the quantitative indicator of the grown colonies of microorganisms (lg CFU/g) were used for the convenience of material presentation and mathematical and statistical processing.

To calculate the alpha-diversity of the gut microbiota, we used the Shannon H′ and Simpson 1/D indices. The Shannon H′ index was calculated using the formula H′ = −∑ pi ln(pi), where pi represents the proportion of individuals attributed to each genus within the gut microbiota. The Simpson 1/D index was calculated using the formula 1/D = ∑ pi^2^, where pi represents the proportion of individuals attributed to each genus within the gut microbiota. We used the Abundance Curve Calculator by Dr. James A. Danoff-Burg and X. Chen, 27 April 05, to calculate the diversity.

In addition, we collected data on several clinical parameters, including LoS, CRP levels, and NLR.

### 4.3. Statistical Analysis

The statistical methods used in the analysis were chosen based on the research questions and the types of data collected. The data were analyzed using GraphPad Prism (version 9), jamovi (version 2.2.5) and JMP 17. Continuous variables were checked for normality using the Shapiro–Wilk test and descriptive statistics were reported as mean ± standard deviation. Categorical variables were reported as percentages.

To compare alpha diversity indices between groups, we used a non-parametric test, the Kruskal–Wallis test, followed by the Dwass–Steel–Critchlow–Fligner (DSCF) post hoc test. This test was chosen due to the non-normal distribution of the alpha diversity data. Alpha diversity indices were calculated using the vegan package in R, which included Shannon, Simpson, and Chao1 indices. The DSCF post hoc test was used to compare all pairs of groups, correcting for multiple comparisons. Spearman rank correlation was used to explore the correlation between LoS, CRP, NLR, and diversity indices. This test was chosen as it does not require the assumption of normal distribution of the data and is appropriate for non-parametric data.

To investigate the relationship between COVID-19 with T2D and COVID-19 without T2D, we used binary logistic regression. The independent variables included in the model were the alpha diversity indices. We calculated odds ratios and 95% confidence intervals, and set statistical significance at *p* < 0.05. All statistical tests were two-tailed, and statistical significance was set at *p* < 0.05.

## 5. Limitation

Although our study contributes to the growing body of literature on the impact of COVID-19 and T2D on gut microbiota diversity and the potential association between gut microbiota diversity and clinical parameters, there are some limitations to our study. Firstly, our study acknowledges that the sample size was relatively small, which may limit the generalizability of our findings. Secondly, the observed differences in alpha-diversity may be influenced by several factors such as diet, age, and disease severity, which were not controlled for in our study. Additionally, the study was limited by the lack of a control group of patients with T2D who did not have COVID-19.

Regarding the methodology used, the culture-based method has several strengths that make it a useful tool for analyzing the gut microbiota. One of the main advantages of this method is that it is relatively inexpensive and straightforward to perform, making it accessible to researchers with limited resources. However, the culture-based method also has some weaknesses that need to be considered when interpreting its results. One limitation is that it may not capture the full diversity of the gut microbiota since some bacterial species may not grow under laboratory conditions. Additionally, the culture-based method may be biased towards the growth of certain bacterial species or groups, which could affect the composition of the microbiota. As a result, sequencing-based methods may provide more comprehensive information on the microbial composition and diversity of the gut microbiota.

Another limitation of our study is the lack of information on the dietary habits of the participants. Diet can play an important role in shaping the composition and diversity of the gut microbiota, and the absence of dietary data may have influenced our findings. Future studies should consider collecting data on dietary intake and controlling for its potential confounding effects. 

## 6. Conclusions

In conclusion, our study demonstrates that metformin therapy is associated with an increase in gut microbiota alpha-diversity in COVID-19 patients with T2D. These findings suggest a potential positive impact of metformin on the microbial composition in this patient group. Further research is needed to understand the underlying mechanisms and explore the clinical implications. Optimizing the gut microbiota through metformin therapy may have implications for personalized treatment strategies and improved outcomes in COVID-19 patients with T2D.

## Figures and Tables

**Table 1:** Demographic characteristics of COVID-19 patients by study group.

Group	Age (Mean ± SD)	Male (%)	Female (%)
Delta variant	55.1 ± 11.4	53.4	46.6
Omicron variant	56.7 ± 4.9	46.6	53.4
Antibiotic-treated group	55.3 ± 11.8	46.6	53.4
Non-antibiotic-treated group	43.6 ± 9.7	40.0	60.0
COVID-19 without T2D	55.4 ± 5.0	53.4	46.6
COVID-19 with T2D	61.5 ± 8.0	53.4	46.6
Metformin-treated patients with T2D and COVID-19 with antibiotic treatment	55.2 ± 5.4	53.4	46.6
Metformin-treated patients with T2D and COVID-19 without antibiotic treatment	50.1 ± 6.6	40.0	60.0

**Table 2:** Alpha diversity indices (Shannon H′ and Simpson 1/D) among eight groups with means and standard deviations (SD).

Group	Simpson 1/D (Mean ± SD)	Shannon H′ (Mean ± SD)	LoS (Mean ± SD)	CRP (Mean ± SD)	NLR (Mean ± SD)
Delta variant	13.2 ± 1.19	2.4 ± 0.09	12.6 ± 2.47	5.1 ± 1.54	5.8 ± 2.47
Omicron variant	13.3 ± 0.40	2.4 ± 0.16	9.1 ± 1.56	2.8 ± 0.72	2.4 ± 0.66
Antibiotic-treated group	11.7 ± 0.70	2.2 ± 0.17	16.0 ± 3.54	7.5 ± 2.47	11.9 ± 3.79
Non-antibiotic-treated group	13.1 ± 0.47	2.5 ± 0.09	9.4 ± 1.76	3.4 ± 0.97	4.6 ± 1.26
COVID-19 without T2D	13.2 ± 0.37	2.4 ± 0.12	9.1 ± 1.59	2.8 ± 0.71	2.4 ± 0.64
COVID-19 with T2D	12.5 ± 0.62	2.3 ± 0.15	16.6 ± 1.84	9.5 ± 0.73	14.7 ± 7.95
Metformin-treated patients with T2D and COVID-19 with antibiotic treatment	12.2 ± 0.39	2.2 ± 0.21	12.7 ± 1.33	6.0 ± 0.38	7.3 ± 0.45
Metformin-treated patients with T2D and COVID-19 without antibiotic treatment	13.0 ± 0.48	2.6 ± 0.23	9.9 ± 1.09	3.8 ± 0.89	3.07 ± 0.57

## Data Availability

Data is contained within the article.
